# Ultraviolet 365 as an Alternative Light Source for Detection of Blood Serum

**DOI:** 10.1111/1556-4029.14439

**Published:** 2020-04-28

**Authors:** Kelly P. Kearse

**Affiliations:** ^1^ Knoxville Catholic High School Knoxville TN

**Keywords:** ultraviolet, blood serum, clotting, alternative light source, plasma, UV

## Abstract

The use of alternative light sources (ALS) in bloodstain analysis has focused on dried (whole) blood, while information on detection of blood serum is lacking. Serum detection by ALS could provide valuable information at a crime scene, as serum may become separated from blood during clotting and cast off, especially in cases where the victim is moved. Additionally, a perpetrator may concentrate on the removal/scouring of dried blood with small amounts of serum going unnoticed, as it dries relatively clear on certain objects. In this report, the detection of human blood serum was evaluated using ultraviolet (UV) light at two different wavelengths. These results show that ultraviolet (UV) at 365 nm (UV365) was effective in the detection of even small amounts of blood plasma and serum, compared with UV at 395 nm, which was not. UV365 was also found to be useful in distinguishing blood imprints from clotting blood which had been transferred to material versus blood that had been added directly. Taken together, these results demonstrate that UV365 may be utilized as a simple, nondestructive method for blood serum detection.

Whole blood consists of two major fractions: a cellular portion, which is approximately 45%, and a liquid portion, plasma, approximately 55%. Plasma is a protein‐rich solution in which the formed elements of blood (white blood cells, red blood cells, and platelets) are suspended (1). Following injury, various blood clotting enzymes function in a cascade fashion to help blood to coagulate; serum is the fluid that remains following separation of a blot clot from whole blood, and unlike plasma, is devoid of the fibrinogens and clotting factors (1). Numerous circumstances may influence the clotting and drying time of blood *ex vivo*, including blood volume, relative humidity and temperature, and surface material (2).

The use of ALS in bloodstain analysis is primarily useful for providing better visualization on certain materials; Polilight, for example, can be effective in detecting bloodstains at 415 nm, particularly those that have been masked over by paint (3, 4). Springer et al. have reported detection of bloodstains on certain materials using lower wavelengths, ~266 nm (5). As an alternative to ALS, bloodstains may be treated with compounds such as luminol or BLUESTAR®FORENSIC to produce a transient blue chemiluminescence which can be documented photographically (6, 7).

In using ALS in the evaluation of blood, the emphasis is placed on detection of whole blood (dried bloodstains). In searching the literature for information about detection of blood serum with ALS, and the distinction of clotting blood transferred to material versus that which had been added afterward, it was noted that relatively little information exists on these topics. Indeed, most reviews on evaluation of body fluids using UV highlight its usefulness for visualization of saliva and semen, with no mention of blood serum (7, 8, 9). Here, the fluorescent detection of blood serum was evaluated using UV at different wavelengths, for example, 365 nm versus 395 nm. These data show that serum fluorescence was masked in whole blood and, however, was revealed upon blood separation by centrifugation or clotting. In addition, experiments were performed to compare the characteristics of clotting blood that is transferred to material versus whole blood that is directly added. The implications of these findings in blood detection and analysis are discussed.

## Materials and Methods

### Blood, Plasma, and Serum

Human blood was obtained from healthy volunteers by the finger‐stick method using a Health Lancing device (CVS pharmacy®, USA) fitted with a microlancet (CVS pharmacy®, USA). Blood was dropped onto Parafilm® M Laboratory Film (Bemis Company, Inc., Oshkosh, WI) and transferred to a 1.5 mL Eppendorf® microcentrifuge tube (Eppendorf, Hamburg, Germany) using a 10–100 µL Eppendorf® micropipettor (Hamburg, Germany), with the volume set at 100 µL; blood was then added to material as needed, using the same micropipettor with the volume set at 20 µL. For clot transfer experiments, blood was transferred from the Eppendorf tube to a fresh piece of Parafilm® M Laboratory Film, or to skin, using an Eppendorf® micro pipettor. After a certain amount of time, filter paper or cloth was placed directly onto the blood pool with very slight pressure. Twenty microliter of blood was typically used for each group. For purification of blood plasma and cellular fractions, 400 µL of whole blood was placed in a 1.5 mL Eppendorf tube and spun in a microcentrifuge (Qualitron Inc., Gyeonggi‐Do, South Korea) for 1 min immediately after collection. The supernatant was removed and transferred to a new tube; this cycle was repeated 2× until no red material was visible. Purification of blood serum was performed in an identical manner, except that blood was first allowed to clot for at least 2 h prior to centrifugation.

### Ultraviolet Light Sources

The UV light systems used in these studies were as follows: a LED UV flashlight, 395 nm, 00F‐51UV‐001A (Esco‐Lite, Shenzhen, China), referred to as Esco‐Lite 395; a handheld UV lamp, 365 nm, UVP UVL‐4FUV Lamp (Analytik Jena, Upland, CA), referred to as Analytik Jena 365; a LED UV flashlight, 365 nm, LED‐UV301‐356 nm (Shenzhen Lightfe Light Limited, Shenzhen, China), referred to as Shenzhen Lightfe 365; and a LED portable 3 watt UV light bulb, 365 nm, UV‐3W‐365UV‐E27‐AC (Golden Gadgets, South El Monte, CA), referred to as Golden Gadgets 365. Photographs were taken using a Sony RX100 digital camera.

### Fabrics

Specific fabrics that were used in this study include a black leather wallet (make/company unknown); a Nike® (Beaverton, Oregon) white leather tennis shoe; a black nylon backpack, the Wanderer style by Altura Photo (Florida, USA); linen that was handwoven from natural, unprocessed flax (Vavstuga, Shelburne Falls, MA) in a 3:1 herringbone pattern by professional weaver Tess Farley (Columbia, SC); Whatman® 1 and 3 mm filter paper (Whatman, Maidstone, Kent, UK); and a blue/green/black patterned 100% silk tie treated to be stain resistant from the manufacturer (Croft & Barrow®: Kohl’s, Menomonee Falls, WI). All fabrics were obtained from the author’s personal collection.

## Results

### UV Detection of Blood Components

**FIG. 1:**
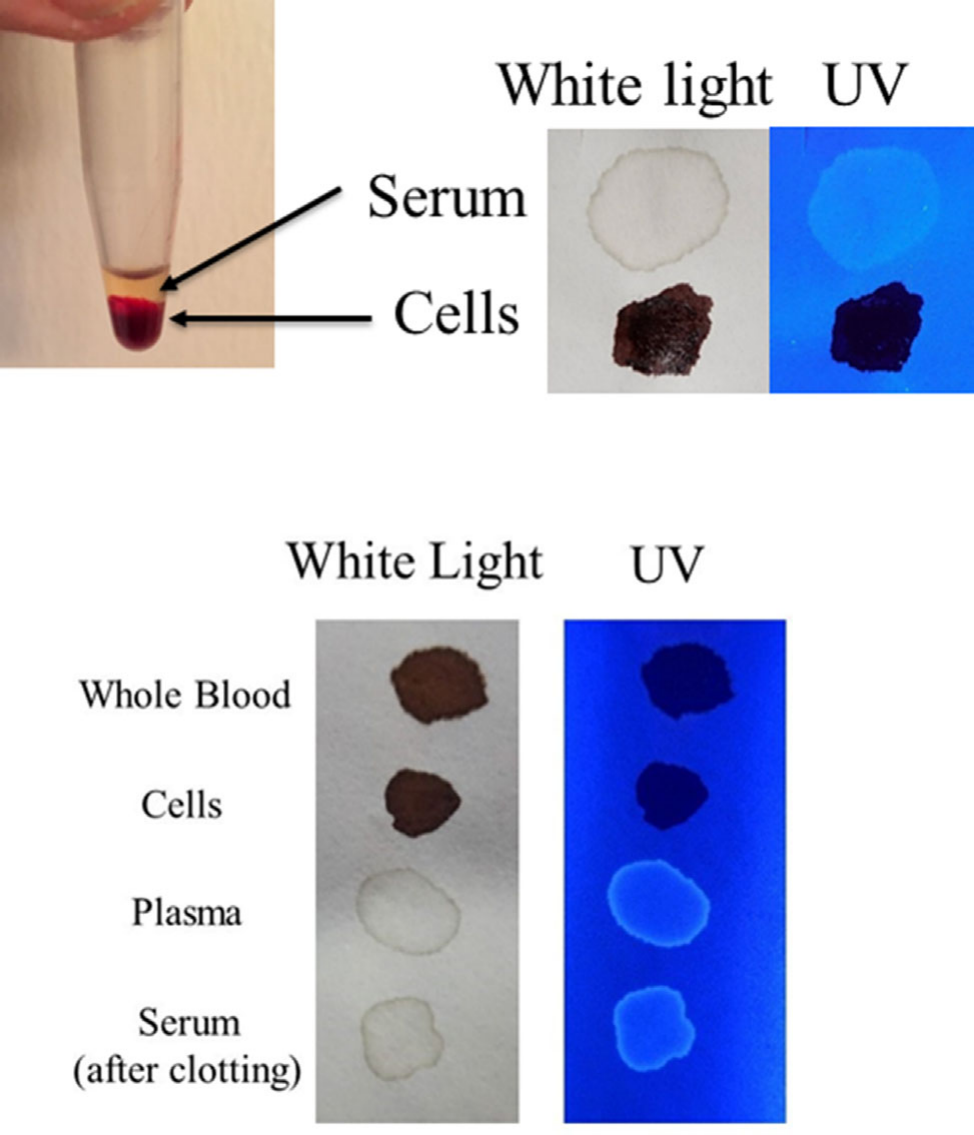
Top: Whole blood and serum were separated by centrifugation into fractions and transferred to filter paper. Samples were evaluated using (normal) white light or UV365 (Analytik Jena 365). Bottom: Same as above, except that whole blood (no separation) and plasma (preclotting) were included [Color figure can be viewed at wileyonlinelibrary.com]

**FIG. 2:**
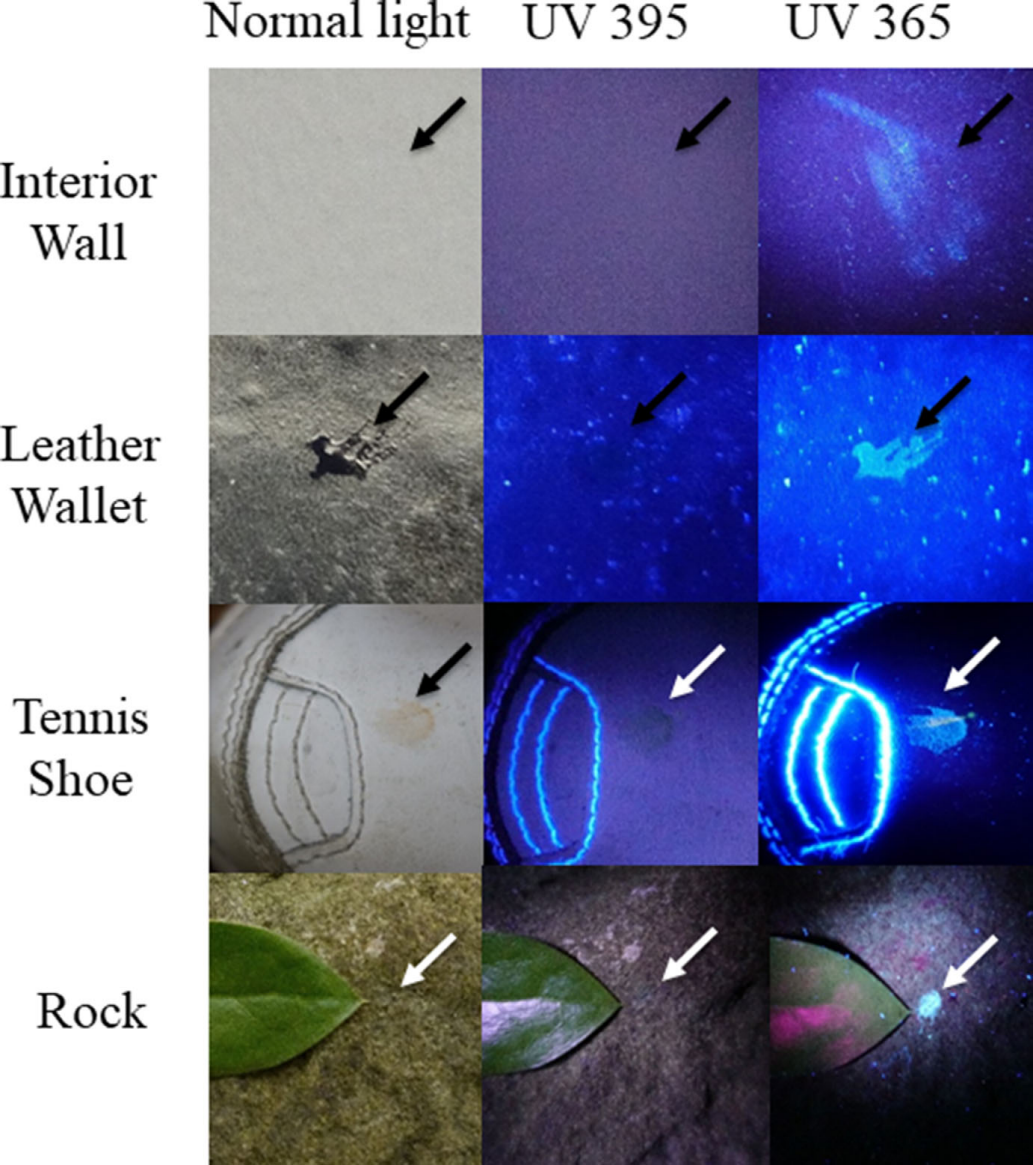
Serum detection on various objects using UV395 and UV365. Approximately 5 µL of serum was added to various articles and examined under normal (white) light, UV395 (Esco‐Lite 395), and UV365 (Shenzhen Lightfe 365). Arrow indicates the position of serum. Note that serum on interior wall and rock samples were difficult to see under normal light and UV395, but effectively detected using UV365 [Color figure can be viewed at wileyonlinelibrary.com]

**FIG. 3:**
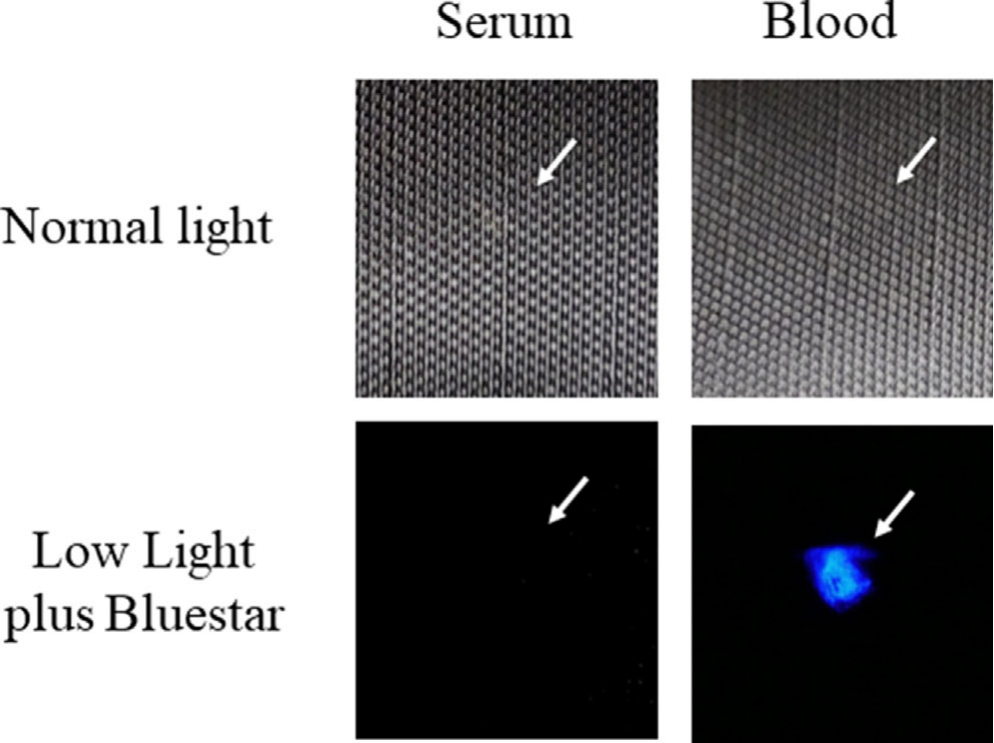
Detection of blood and serum using BLUESTAR®FORENSIC**.** Approximately 5 µL of blood or serum was added to a black nylon backpack and evaluated using normal light (untreated) or under low light following addition of BLUESTAR®FORENSIC. Arrow indicates the position of serum (left) or blood (right) [Color figure can be viewed at wileyonlinelibrary.com]

Initially, the detection of blood plasma and serum purified by microcentrifugation was examined using a handheld UV fluorescent lamp (Analytik Jena 365). As shown in Fig. 1, whole blood and the cellular fraction appeared relatively dark under UV, whereas detection of serum and plasma was enhanced (Fig. 1). By visual inspection, fluorescence of blood plasma and serum appeared to be of relatively similar intensities (Fig. 1). Next, the fluorescence of serum applied to various articles was evaluated using LED flashlights at two different wavelengths, UV365 and UV395. As demonstrated, serum was essentially invisible under normal light or UV395 on certain objects (interior wall, rock), but was revealed using UV365 (Fig. 2). Unlike whole blood, serum was not effectively detected using BLUESTAR®FORENSIC (Fig. 3). Taken together, these results demonstrate that UV365 is useful for the detection of blood serum on a variety of substances.

### Development and Detection of Serum Halos/Edges

**FIG. 4:**
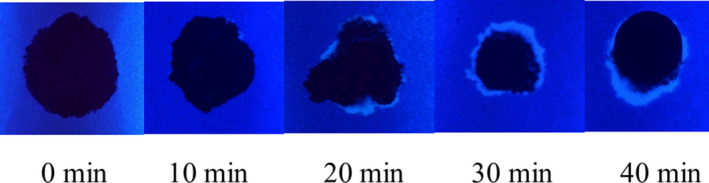
Time dependence of serum halos/edge detection using ultraviolet light**.** Whole blood was placed on Parafilm® M Laboratory Film and allowed to clot for the time period indicated before contact with filter paper; the filter paper was removed after drying, and samples evaluated using UV365 (Analytik Jena 365). Note the time‐dependent formation of a serum halo/edge at the periphery of the bloodstain [Color figure can be viewed at wileyonlinelibrary.com]

**FIG. 5:**
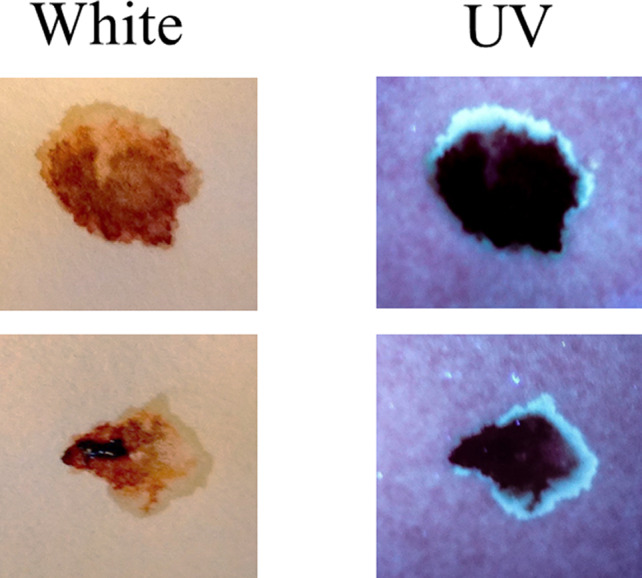
Detection of serum halos/edges on clotting blood transferred from skin**.** Whole blood was placed on skin and allowed to clot for 45 min before contact with filter paper; the filter paper was removed after drying, and samples evaluated using UV365 (Shenzhen Lightfe 365) [Color figure can be viewed at wileyonlinelibrary.com]

**FIG. 6:**
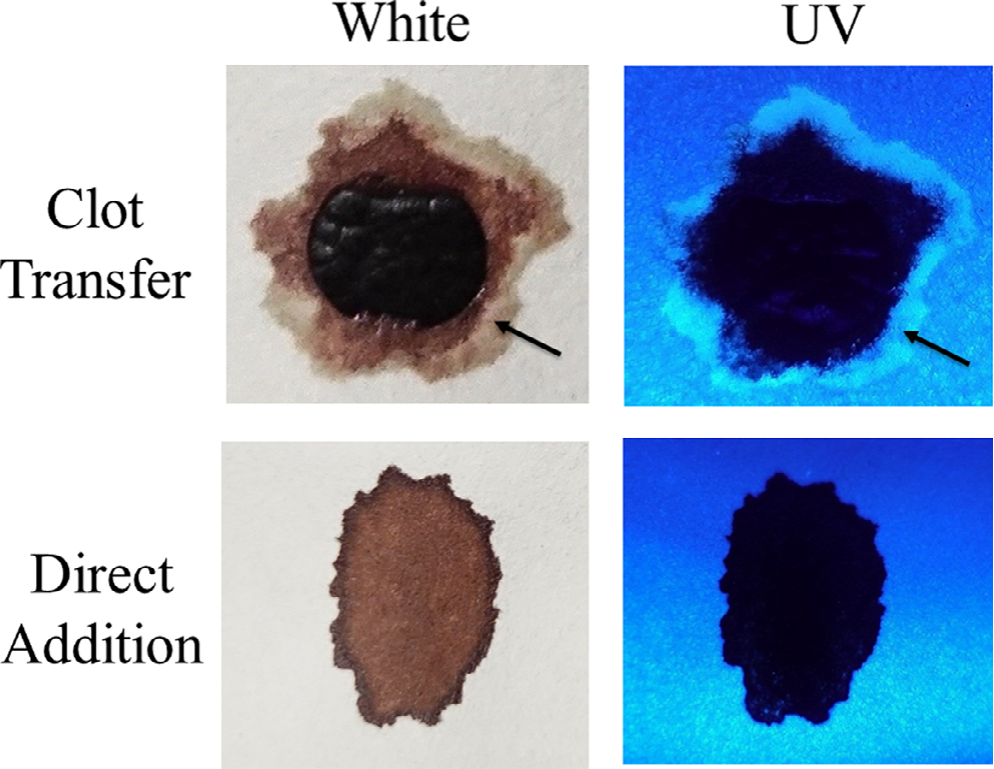
UV detection of serum halos/edges in blood samples transferred before or after clotting**.** Top: Whole blood was placed on Parafilm® M Laboratory Film and allowed to clot ~40 min before contact with filter paper; the filter paper was removed after drying, and samples evaluated using UV365 (Analytik Jena 365). Arrow indicates serum halo/edge at the periphery of the bloodstain. Bottom: Whole blood was added to filter paper, allowed to dry, and samples evaluated using UV365 (Analytik Jena 365). Note the absence of a serum halo/edge when fresh blood was added directly [Color figure can be viewed at wileyonlinelibrary.com]

**FIG. 7:**
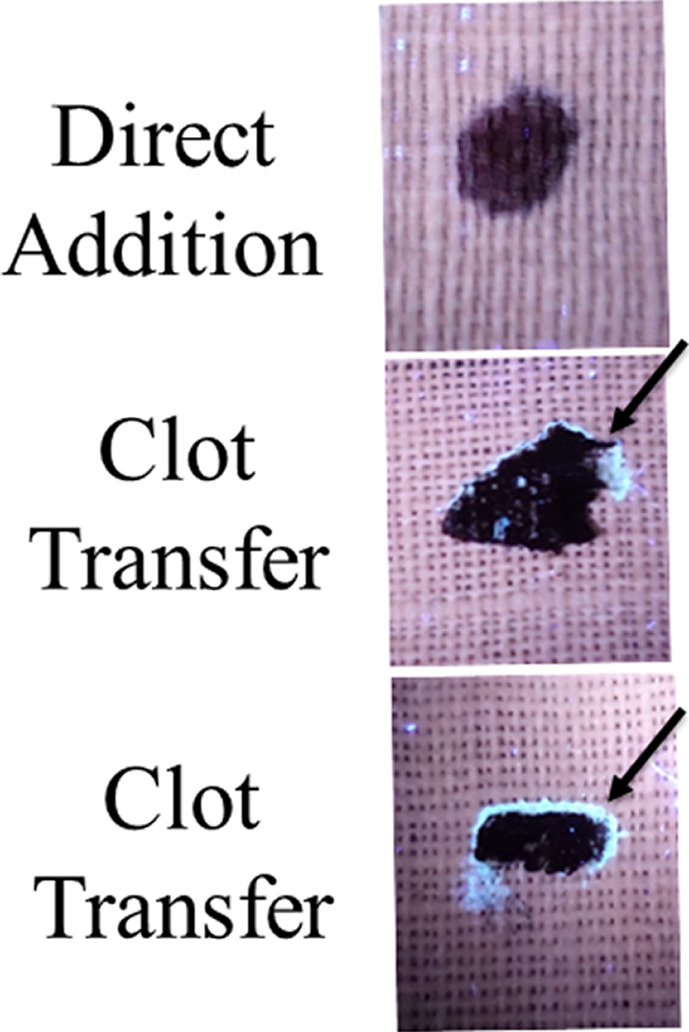
UV detection of serum halos/edges in blood samples transferred before or after clotting. Top panel: Whole blood was directly added to linen, allowed to dry, and samples evaluated using UV365 (Golden Gadgets 365). Note the absence of a serum halo/edge when blood was added directly. Alternatively, whole blood was placed on Parafilm® M Laboratory Film and allowed to clot 45 min before contact with linen; linen was removed after drying, and samples evaluated using UV365 (Golden Gadgets 365), (middle and bottom panels). Arrow indicates serum halo/edge at the periphery of the bloodstain [Color figure can be viewed at wileyonlinelibrary.com]

**FIG. 8:**
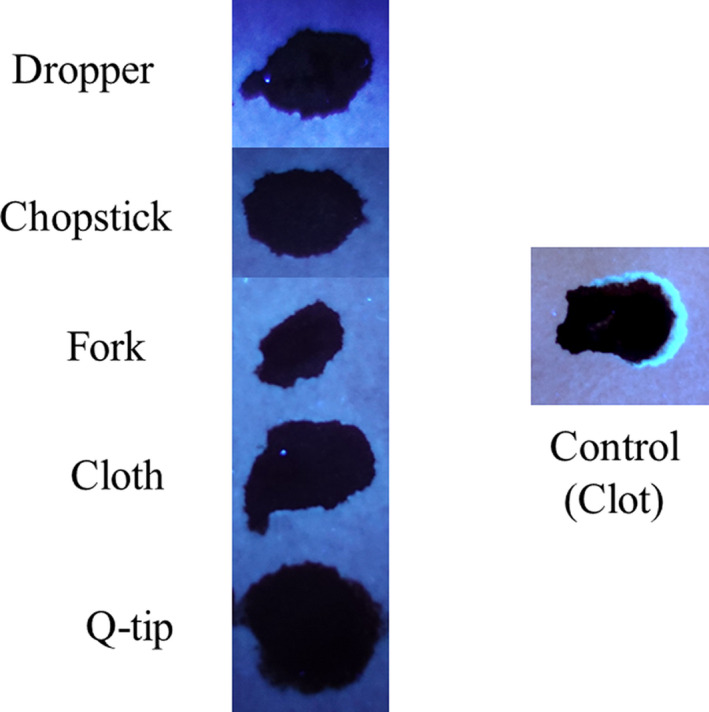
Evaluation of serum halos/edges in blood added to filter paper using different applicators**.** Blood was added to filter paper right after collection using the indicated applicator. After drying, samples were examined using UV365 (Shenzhen Lightfe 365). Note the absence of a serum halo/edge at the periphery of the bloodstain, unlike transfer of clotting blood (right‐hand side) [Color figure can be viewed at wileyonlinelibrary.com]

**FIG. 9:**
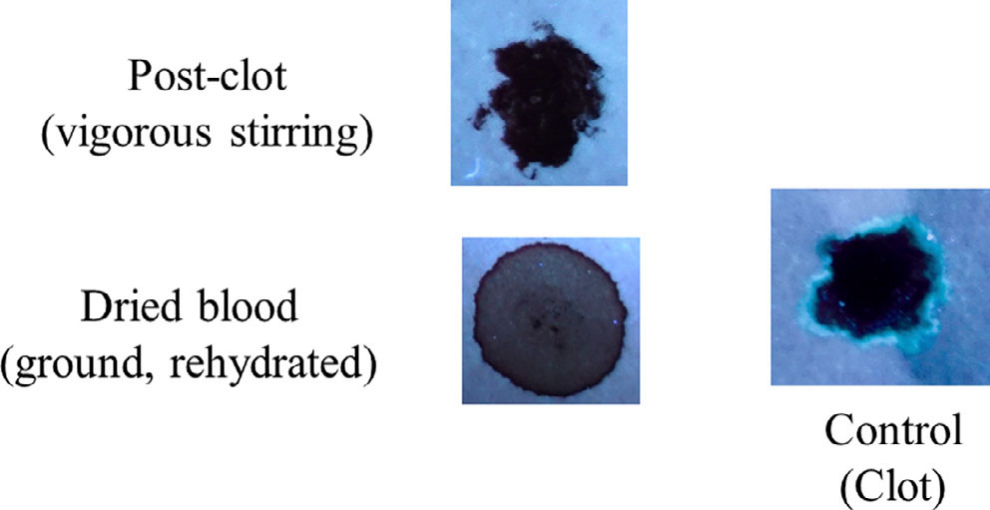
Evaluation of serum halos/edges in blood added postclotting Top: Blood was allowed to clot for several hours and the clot disrupted by vigorous stirring. Blood was then transferred to filter paper, and after drying, samples were examined using UV365 (Shenzhen Lightfe 365). Note the absence of a serum halo/ring at the periphery of the bloodstain, unlike what is observed with transfer of clotted blood (right). Bottom: Clotted blood that had dried for several days was ground using a mortar and pestle, rehydrated with a small volume of distilled water, and added to filter paper. After drying, samples were examined using UV365 (Shenzhen Lightfe 365). Note the absence of a serum halo/ring at the periphery of the bloodstain [Color figure can be viewed at wileyonlinelibrary.com]

Next, the development and detection of serum “halos” or edges, which may form at the periphery of the bloodstain during the clotting process was evaluated. Of particular interest was to determine whether serum edges were specific for clotting blood that was transferred, or whether these could also be observed when blood was added directly to material. The appearance of serum edges in blood imprints was time‐dependent (Fig. 4); in this system using relatively small volumes of blood (~25 µL), it was noted that after 50–60 min, only minimal transfer of bloodstains was observed (data not shown). Serum edges were also detected when blood was placed onto skin, and clotting allowed to proceed prior to transfer (Fig. 5). Unlike what was observed with imprints of clotting blood, when blood was added directly to material, no serum edges were apparent using either filter paper or linen (Figs 6 and 7). These results were confirmed using a variety of implements to apply blood, as shown in Fig. 8. Blood that had clotted for several hours was vigorously stirred and added to filter paper, with no serum edge being visible (Fig. 9); when dried blood that was several days old was ground in a mortar and pestle, rehydrated, and added to material, a similar result was obtained (Fig. 9). Collectively, these results demonstrate that blood added directly to filter paper or linen did not result in the presence of a serum edge, detectable by UV light. Serum edges were only observed when blood was imprinted during the clotting process.

**FIG. 10:**
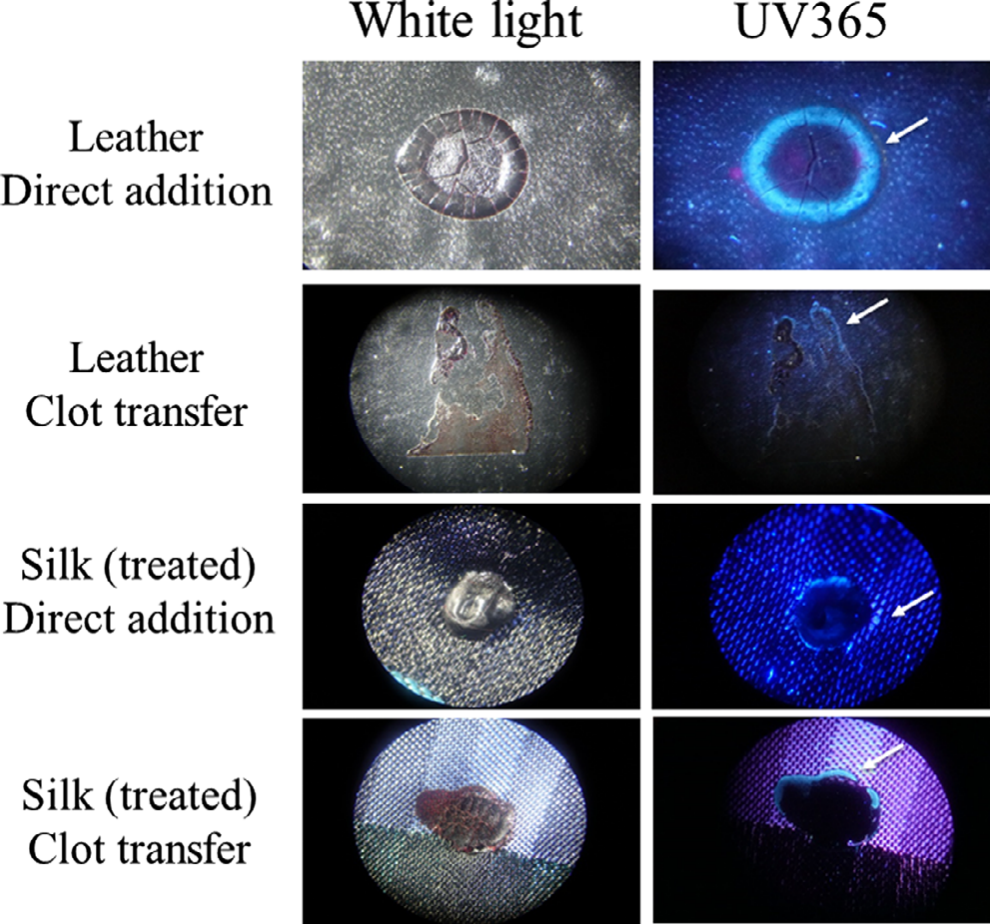
Evaluation of serum halos/edges in blood added pre‐ and postclotting. Whole blood was placed on the indicated article (direct addition) or placed on Parafilm® M Laboratory Film and after 45 min transferred to the article (clot transfer). After drying, samples were evaluated using a stereomicroscope under white light or UV365 (Golden Gadgets 365). Serum edges are indicated by arrow [Color figure can be viewed at wileyonlinelibrary.com]

Linen is described as the world’s strongest natural fiber and, like filter paper, is quite absorbent, with linen able to retain approximately 20% of its own weight (10). To evaluate properties of serum edges on less absorbent material, a similar set of experiments was performed using a silk tie, which had been treated during manufacture to become stain resistant, and a leather wallet. Samples were evaluated under a stereomicroscope using both normal (white) light and UV365. As shown, a serum halo was detectable on blood that had been directly added to leather, with the bloodstain maintaining a round shape (Fig. 10). A serum edge was also present in clot transfer samples on leather, although more spread out, which followed the irregular shape of the stain (Fig. 10). As with leather, a serum edge was observed in both direct addition and clot transfer samples using treated silk (Fig. 10). Thus, it is possible to observe a serum halo/edge when blood is directly added, depending upon the surface of the material.

**FIG. 11:**
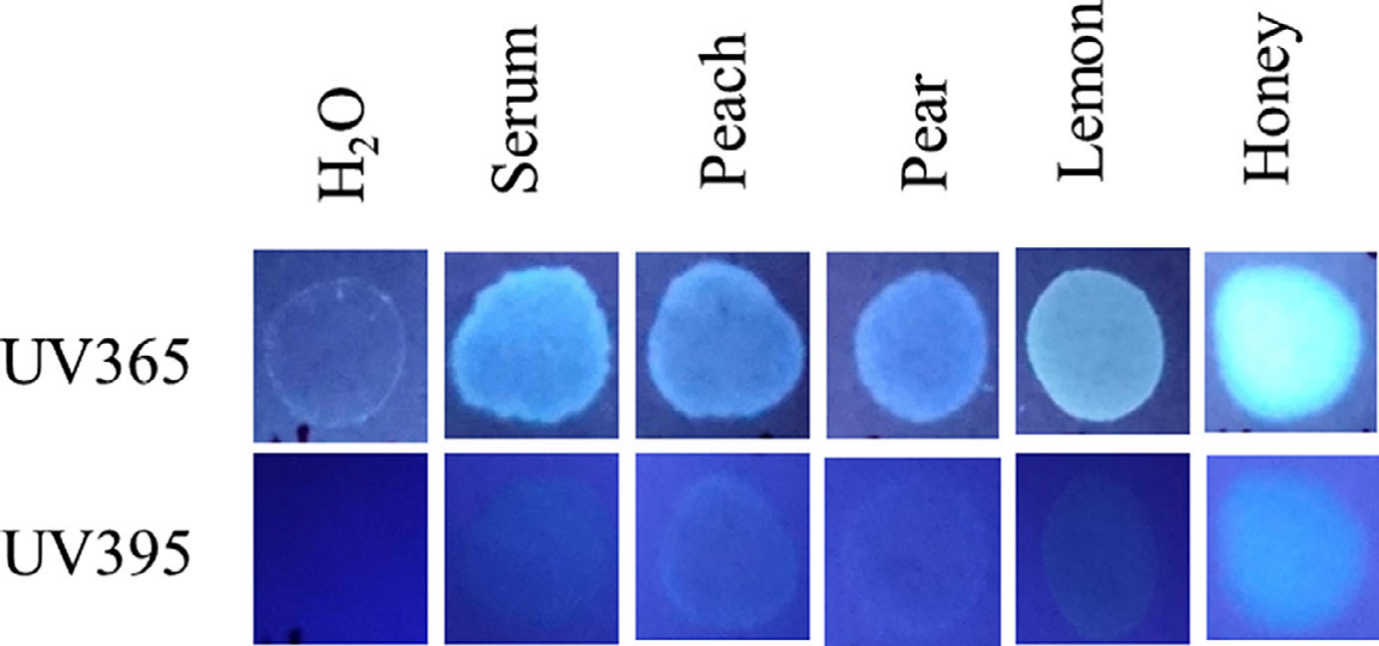
Fluorescence of honey and various fruit juices using UV365 and UV395. Approximately 10 µL of water (control), serum, honey, or various fruit juices was added to filter paper and evaluated under UV365 (Shenzhen Lightfe 365) or UV395 (Esco‐Lite 395). All juices were freshly squeezed [Color figure can be viewed at wileyonlinelibrary.com]

**FIG. 12:**
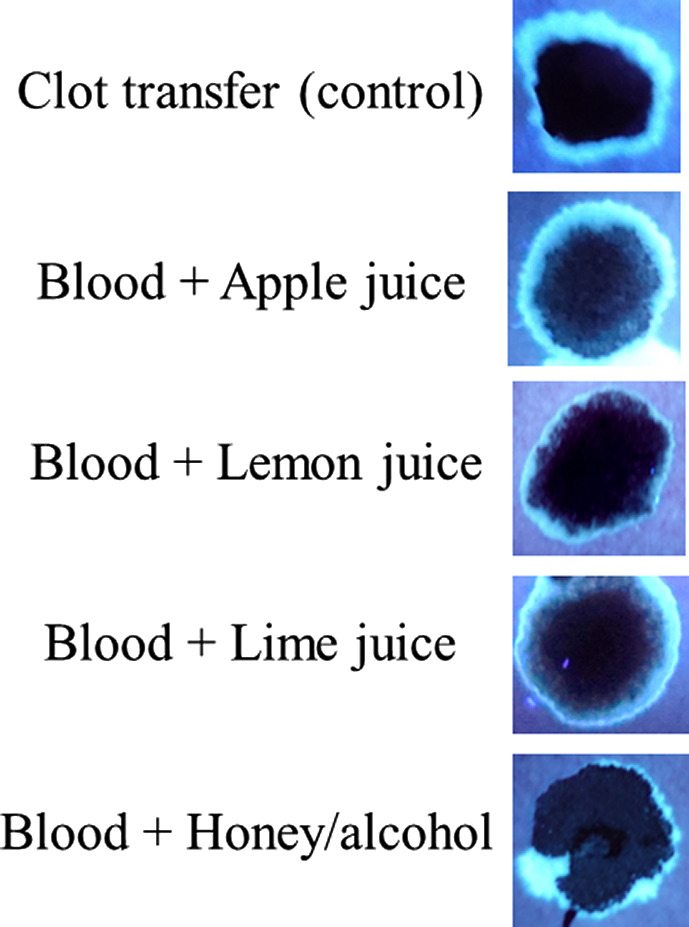
UV fluorescence of blood plus additives (direct addition). Blood plus the indicated additive were placed directly onto filter paper, and after drying, samples were evaluated using UV365 (Shenzhen Lightfe 365). The approximate ratio used was 1:5 of additive to blood, respectively. In the honey/alcohol sample, neat honey was diluted 1:4 in 70% isopropyl alcohol prior to its use. Note the presence of a fluorescent halo/edge in blood samples containing additives. Top panel shows a sample from a clotting imprint (control) [Color figure can be viewed at wileyonlinelibrary.com]

Finally, experiments were done to determine, if under any circumstances, a fluorescent halo/edge might be present in blood samples added directly to paper. As shown in Fig. 11, like serum, juice from multiple fruits fluoresced under UV365, (Fig. 11), as did (undiluted) honey, which was particularly bright (Fig. 11). To determine whether bloodstains containing serum halos/edges could be mimicked by the addition of such substances, blood mixtures were created and added directly to filter paper. As demonstrated in Fig. 12, serum halos/edges were formed that were similar in appearance to those observed in clotting imprints (Fig. 12). Taken together, these results illustrate that juices from certain common fruits exhibit fluorescence, which could result in an inaccurate determination of serum detection with UV365. In addition, these data show that serum halos/edges can be mimicked by direct addition of blood to filter paper, with the inclusion of certain additives.

## Discussion

As demonstrated in this report, blood plasma and serum were effectively detected using UV365 on a variety of materials. Three different sources of UV365 illumination were utilized, including a fluorescent lamp, a LED flashlight, and a LED light bulb, with relatively similar results. UV detection of serum could prove valuable in cases where a victim is moved during the blood clotting process, and separated serum may adhere to adjacent surfaces. Moreover, upon drying, small amounts of serum may go unnoticed by a perpetrator that is more focused on trying to clean up the major, more obvious bloodstains. As shown in these studies, even very small volumes of serum (~5 µL) can be effectively detected with UV365. UV365 could be utilized as a simple, nondestructive method in the initial examination of a crime scene; confirmation that serum was indeed present would, of course, require further testing, similar to evaluation for the presence of semen or saliva using ALS.

The current study provides data concerning the formation and detection of serum halos/edges in a microscale environment. The use of UV365 could potentially prove useful in certain cases for the discrimination of articles to which blood has been added after the fact versus those to which clotting blood had been transferred during the initial event. While the results in the current study support the idea that when adsorbent material was used, serum halos/edges were only observed during imprinting of clotting blood, it was also demonstrated that such “contractile rings” could be mimicked when blood containing various additives was applied directly. This is an important consideration in the overall determination if clotting blood was indeed transferred to a fabric. In addition to absorbency, the intensity of background fluorescence of fabrics can also affect serum visualization. For example, we found that detection of serum on jean material and white cotton T‐shirts was relatively difficult (data not shown). Thus, serum detection using UV365 may be more suitable for certain materials than others. It would also be interesting to evaluate serum detection using other methods, Polilight, for example, and in areas that have been painted over or cleaned with various solvents, particularly those that are not water‐soluble. Future efforts will involve investigation using increased blood volumes and evaluation of a variety of textiles and other articles, including parameters that could affect certain surface properties.
